# Fine Detection Method of Strata Information While Drilling—From the Perspective of Frequency Concentrated Distribution for Torque

**DOI:** 10.3390/s25175563

**Published:** 2025-09-06

**Authors:** Jingyi Cheng, Xin Sun, Zhijun Wan, Xianxin Zhang, Keke Xing, Junjie Yi

**Affiliations:** 1 School of Mines, China University of Mining and Technology, Xuzhou 221116, China; 5759@cumt.edu.cn (J.C.); zhjwan@cumt.edu.cn (Z.W.); ts23020072a31tm@cumt.edu.cn (X.Z.); tb20020032b4@cumt.edu.cn (K.X.); ts23020066a31@cumt.edu.cn (J.Y.); 2 Key Laboratory of Deep Coal Resource Mining (CUMT), Ministry of Education of China, School of Mines, China University of Mining & Technology, Xuzhou 221116, China

**Keywords:** strata strength, measurement while drilling (MWD), frequency concentrated distribution, characteristic interval, peak torque

## Abstract

Measurement while drilling technology (MWD) has emerged as a pivotal approach for geological exploration. However, the accuracy of existing geological recognition models remains limited, primarily due to data fluctuations that result in high overlap rates and reduced reliability of drilling parameters. This study takes torque data as an example and analyzes the frequency distribution laws of torque responses across rock with varying strengths. A quantitative model of the frequency distribution characteristic interval is established, and a rock information prediction approach based on frequency distribution characteristics is proposed. The results indicate that torque frequency distributions for homogeneous rock exhibit a unimodal pattern, whereas those for composite rocks display multimodal characteristics. The boundaries of the frequency distribution characteristic intervals are mathematically defined as CIS = *T_p_*|_(*dF*/*dT*) = 0_ ± *σ* and CIM = *x_li_* ± 0.5*∆x_i_*. The strength prediction model constructed using torque within the characteristic interval achieves an average accuracy of 85.3%. Furthermore, the frequency of torque within the characteristic interval enables the estimation of rock stratum thickness. This research contributes to enhancing the accuracy of rock information identification.

## 1. Introduction

Before mining engineering construction activities, such as roadway excavation and support design, the geological survey is indispensable due to its significant role in identifying the sudden instability of various rock masses caused by the randomness and concealment of local geological changes in strata [1,2]. However, traditional geological detection methods like core drilling and drilling peep have limitations, including poor real-time performance, high cost, limited detection range, and extensive manual involvement [3]. Since the 1980s, scholars have introduced MWD technology into coal mines for geological investigation [4]. Numerous studies have demonstrated the effectiveness of MWD technology in conducting real-time and efficient surveys of geology [5,6,7]. However, existing MWD technology commonly suffers from low identification accuracy. To address this issue, numerous researchers have focused on enhancing the precision of identification models through building new indicators and optimizing algorithms [8,9].

Regarding building new indicators, the drilling specific energy (SED) was defined as the energy required to break a unit volume of rock, and there existed a remarkable correlation between SED and rock properties [10]; the scholars established a functional relationship between SED and rock strength [11], while the SED was also utilized to predict discontinuities, such as joints and fractures within rock masses [12]. Leung et al. [13] identified the limitations of SED in terms of low specificity and high variance; therefore, they processed the SED using a logistic function to obtain modulated specific energy, which effectively distinguished between coal and non-coal strata. Based on dimensional analysis theory, Zhang et al. [14,15] developed a rock drillability index (RDA) and provided corresponding ranges of RDA for different lithologies; a linear regression model was established between the RDA and rock strength. In addition, some scholars use Savitzky–Golay filtering [16], Kalman filtering [17], ensemble empirical mode decomposition [18], and other methods to reduce the noise of drilling parameters, so as to improve the response sensitivity of drilling parameters to rock geology.

In terms of model algorithms, Burak et al. [19] investigated the issue of lithology identification delays caused by sensor offset in logging while drilling, comparing the effectiveness of petrophysical methods, a combination of unsupervised and supervised machine learning approaches, and the random forest (RF) classification algorithm for lithology identification. He found that the RF model significantly improved lithology prediction accuracy by optimizing input parameters. Furthermore, based on the RF algorithm’s prediction of compressional wave velocity and lithology, he proposed a hybrid rate of penetration (ROP) prediction model and demonstrated that parameters such as compressive strength and weight on bit have a significant influence on ROP [20]. Sun et al. [21] developed a decoupling model between control parameters and comprehensive indicators; they predicted rock strength using support vector machines (SVMs) and decoupled comprehensive indicators, and the accuracy of the prediction model rose to 84%, while the relationships between the drilling rate index and physicomechanical rock properties was also confirmed [22,23]. Wang developed a lithology identification model using neural networks based on time-domain and frequency-domain features of drilling tool vibrations. This model enables the recognition of formation changes through the analysis of vibration signals, achieving an average accuracy of 89.57% in lithology classification [24]. Liu et al. [25] compared the identification accuracy of rock properties using BP neural networks and SVM and found that both models achieved an accuracy exceeding 80%. Furthermore, three artificial intelligence models were developed using artificial intelligence tools, such as artificial neural networks (ANNs), adaptive neuro-fuzzy inference system (ANFIS), and support vector machine (SVM), to predict the UCS of the downhole formations while drilling [26]. Li et al. [27] proposed a data cleaning method to correct drilling parameters and enhance their correlation with formation information. By employing a soft voting strategy to integrate classifiers, including SVM, DT, KNN, and neural network, the lithology identification model achieved an accuracy rate of 98.79% in field tests.

The aforementioned analysis indicates that numerous scholars have conducted extensive and beneficial research on the identification of strata strength and structure through MWD technology, significantly advancing the development of MWD technology in coal mining. However, external factors such as drill rig vibration [28], drill rod buckling [29], and rock heterogeneity [30] contribute to significant fluctuations in drilling parameters. Consequently, there is a high overlap rate among drilling parameters, which obscures their response characteristics to differences in rock strength. This issue is particularly prominent in field drilling operations. Furthermore, control parameters (such as penetration rate) exhibit a strong coupling relationship with drilling parameters, where the influence of penetration rate can even surpass that of rock strength [31]. As a result, this becomes a significant factor in reducing the sensitivity of drilling parameters. When various external factors cause low sensitivity between drilling parameters and rock strength, merely building new indicators and optimizing algorithms are insufficient to effectively improve the accuracy of MWD technology. Therefore, it is undoubtedly crucial to find methods for reducing the overlap rate of drilling parameters for different strength strata, while ensuring the reliability of drilling parameters to enhance the accuracy of MWD technology.

From the perspective of drilling parameters throughout the entire borehole, different strata exhibit varying degrees of overlap in their drilling parameters, with a higher overlap rate observed for strata with smaller differences in strength. However, specific concentrated distribution ranges exist for the drilling parameters of each stratum, within which significant differences are observed [32]. In other words, the overlap rate of drilling parameters between different rock layers is significantly reduced within these ranges. By utilizing the drilling parameters within these ranges as a basis for predicting strata strength and structure, the prediction accuracy of strata information can be effectively improved. This study presents an innovative approach to identifying strata information through MWD technology by considering the frequency distribution of drilling parameters, thereby contributing significantly to improving accuracy in identifying strata information.

## 2. Experiments

### 2.1. Device and Materials

The roadways of coal mines commonly consist of strata types such as coal, sandy mudstone, coarse-grained sandstone, and fine-grained sandstone, which collectively make up over 85% of all types of strata. These strata exhibit a strength range from 5 MPa to 60 MPa [33,34]. Therefore, four groups of composite samples with varying strengths were prepared and divided into two layers. Table 1 and Table 2 show the material ratio and strength grade of different rock samples, respectively.

**Figure 1:**
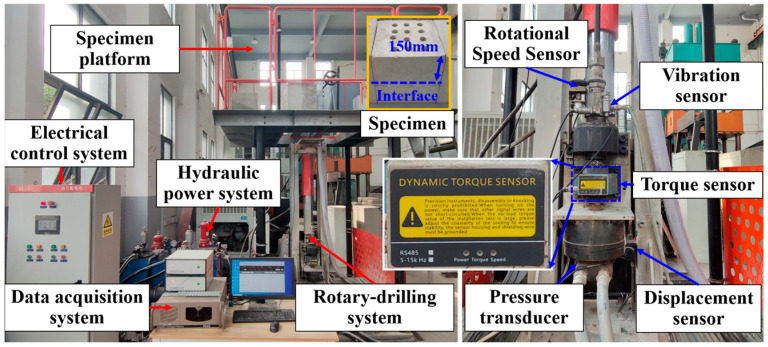
Geological measurement while drilling system (GMWDS).

The dimensions of the rock sample measure 300 mm × 300 mm × 300 mm, with the interface of each composite sample positioned at a distance of 150 mm. During the specimen preparation process, thorough mixing was applied to minimize air bubbles within the specimens in order to reduce heterogeneity of the rock samples. At the same time, a consistent curing environment was maintained to minimize the deviation between the designed and actual strength of the specimens. Utilizing the geological measurement while drilling system (GMWDS), as illustrated in Figure 1, drilling experiments were conducted. The electrical control system maintains constant penetration rate and rotation speed, while the data monitoring system samples at a frequency of 50 Hz, allowing for the monitoring of drilling parameters such as displacement, thrust, and torque. The selected drilling tools include B19 drill rods and Φ28 two-wing PDC drill bits.

The drilling process adopts a “constant rotational speed–constant penetration rate” control mode, and the drilling test plans are as shown in Table 3. Furthermore, it should be noted that all drilling experiments in this study were conducted without applying confining pressure to the rock specimens. For each sample, nine boreholes are drilled, and drilling is stopped when the borehole depth reaches 280 mm. The drilling machine is then reset in preparation for the next drilling operation.

### 2.2. Analysis of Drilling Process

**Figure 2:**
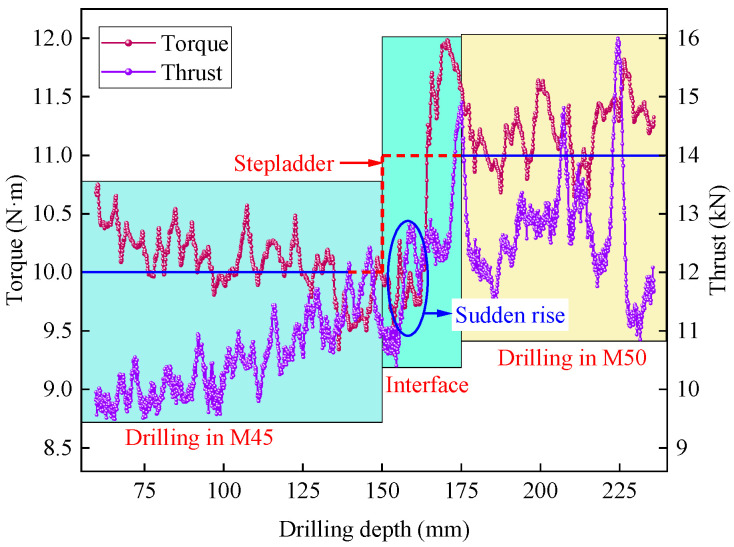
Thrust and torque response characteristics of borehole S4-B4.

Figure 2 shows the thrust and torque curves during the stable drilling phase of the S4-B4 borehole, where the penetration rate and rotation speed are 4 mm/s and 500 rpm, respectively. From the figure, it can be observed that both thrust and torque exhibit a significant increasing trend with the increase in rock strength. Specifically, while the torque remains relatively stable when drilling within the same rock layer, the thrust gradually increases with the drilling depth. When transitioning from M45 to M50 during the drilling process, the torque displays a distinct “step” characteristic, whereas the characteristic of the thrust is not as pronounced.

When the drill bit passes through the rock layer interface (150 mm), both thrust and torque increase rapidly. This is due to the higher energy required to break through the hard rock, which consequently leads to an increase in the thrust and torque output by the drilling machine. The transition phase at the interface lasts approximately 25 mm and takes about 6.2 s. Furthermore, as the drill bit transitions from low-strength rock to high-strength rock, the degree of fluctuation and the rate of fluctuation in the data become significantly more pronounced.

### 2.3. Noise Reduction in Drilling Parameters

**Figure 3:**
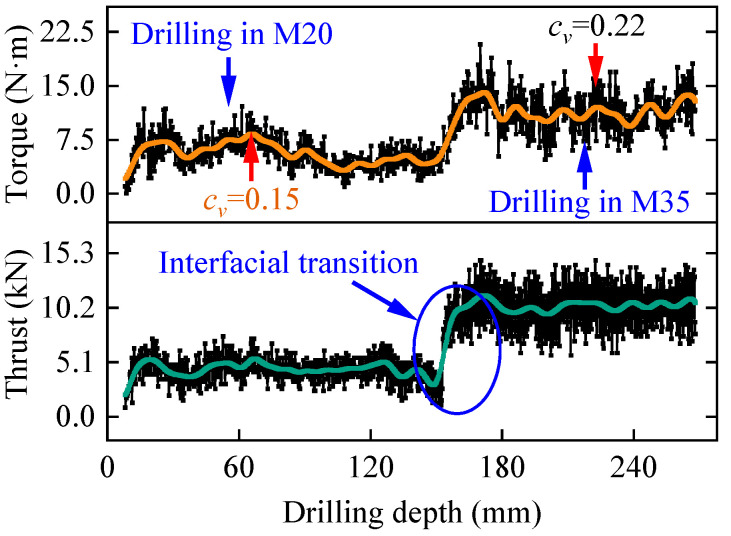
The noise reduction effect of EEMD algorithm.

To address the issue of significant noise in drilling parameters caused by factors such as system vibrations, this study employs the ensemble empirical mode decomposition (EEMD) method for denoising the drilling parameters [35]. The drilling parameters from the S2-B1 borehole were input into the EEMD denoising algorithm, and the comparison curves of the drilling parameters before and after denoising are shown in Figure 3. It can be observed from the figure that the higher the rock strength, the more pronounced the fluctuations in the drilling parameters, indicating that the noise signals contained within the drilling parameters are more abundant. This can be attributed to the higher frequency and greater amplitude of energy exchange between the drill bit and the rock when drilling high-strength rock, which also increases the vibrations of the drill pipes, thereby leading to a greater accumulation of noise signals in the drilling parameters. After applying the EEMD method for denoising, the coefficient of variation of the drilling parameters decreased from 0.22 to 0.15, indicating that the noise signals within the drilling parameters were reduced by approximately 32%. This demonstrates that the EEMD method is effective in reducing the noise signals present in the original drilling parameters.

## 3. Response Characteristics of Torque to Different Rock Strengths

### 3.1. The Impact of Penetration Rate on Torque

**Figure 4:**
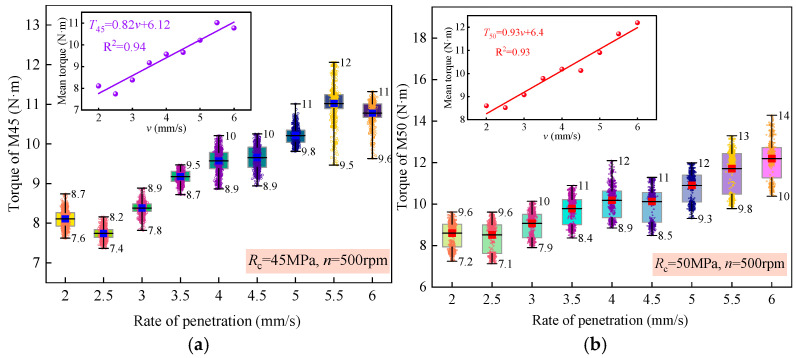
Relationship between penetration rate and torque: (**a**) M45 sample. (**b**) M50 sample.

In the drilling experiments conducted under the “constant rotational speed—constant penetration rate” control mode, in addition to rock strength, the penetration rate also significantly impacts the drilling parameters. Therefore, it is essential to minimize the influence of penetration rate on the drilling parameters to highlight the relationship between rock strength and drilling parameters, which plays a crucial role in improving the accuracy of rock strength identification during drilling. Figure 4 illustrates the relationship between penetration rate and torque during the drilling of sample 4.

Figure 4 indicates that for rock of the same strength, the torques all increase as the penetration rate rises. Specifically, when the penetration rate increases from 2 mm/s to 6 mm/s, the average torque increments for the M45 and M50 samples are 2.7 N·m and 3.6 N·m, respectively. This suggests that the influence of penetration rate on torque becomes more pronounced with higher rock strength, a phenomenon that aligns with the experimental results of scholar Liu [36]. Additionally, the average torque values for the M45 and M50 samples, along with their corresponding penetration rates, were fitted, resulting in the fitting equations presented in Equation (1):

(1) $$\left\{\begin{array}{l} T_{45}=0.82v+6.12(R^{2}=0.94)\\ T_{50}=0.93v+6.40(R^{2}=0.93) \end{array}\right.$$

The average slope of the fitting equation is 0.86, as evident from Equation (1), and the determination coefficients of the two equations are greater than 0.93, indicating a significant linear relationship between penetration rate and torque. With every 1.0 mm/s increase in penetration rate, the torque increases by approximately 0.86 N·m. Previous studies have demonstrated that the relationship between rock strength and torque variation can be roughly expressed as 0.1~0.2 N·m/MPa [37]. Hence, it becomes apparent that the influence of penetration rate on torque surpasses that of rock strength, making it the dominant factor in torque variation, which is clearly unacceptable. Consequently, when utilizing torque to predict rock strength, it is imperative to first eliminate the impact of penetration rate on torque using Equation (2), ensuring the reliability of torque data.

From Equation (1), it can be observed that the average slope of the fitting equation is 0.86, and the coefficient of determination is greater than 0.93, indicating a significant linear relationship between penetration rate and torque. Specifically, for every 1.0 mm/s increase in penetration rate, the torque increases by approximately 0.86 N·m. Previous research has suggested that the relationship between rock strength and the change in torque is roughly in the range of 0.1 N·m/MPa to 0.2 N·m/MPa [37]. This indicates that the impact of penetration rate on torque may even surpass that of rock strength; such a conclusion is clearly unacceptable. Based on the average values of the slope and intercept, Equation (2) was established to eliminate the impact of penetration rate on torque:

(2) $$T_{c}=T_{r}-0.86v+6.3$$

where *T_c_* represents the torque obtained after eliminating the influence of penetration rate, while *T_r_* refers to the original torque, and the variable *v* denotes the penetration rate.

### 3.2. Overlap Characteristics of Torque for Different Rock Strengths

Under the influence of factors such as drilling machine vibrations, rock heterogeneity, and human interference, the torque exhibits considerable fluctuations, resulting in substantial overlap of torque data across rocks of varying strengths. This overlap diminishes the sensitivity of torque response to changes in rock strength. In this study [38], the degree of torque fluctuation is described using the coefficient of variation presented in Equation (3):

(3) $$c_{v}=\sigma /\mu$$

where *c_v_* is the coefficient of variation, *μ* is the mean value of the data, and *σ* is the standard deviation of the data.

**Figure 5:**
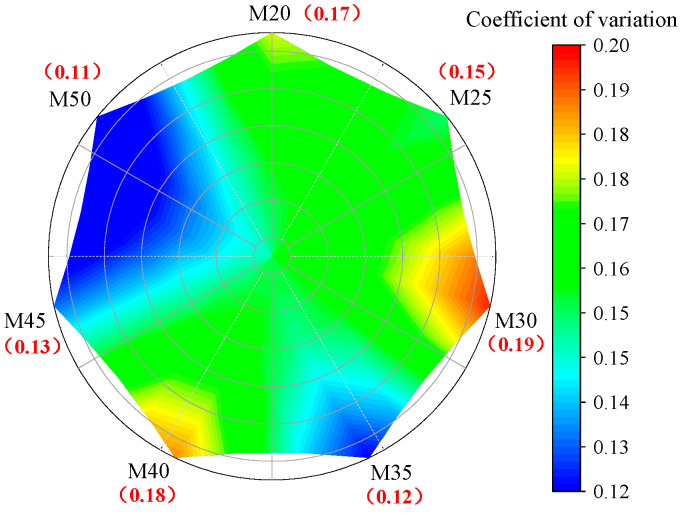
Coefficient of variation of torque under different rock strength.

Figure 5 illustrates the coefficient of variation of torque data for rocks of varying strengths. It can be observed that the distribution range of the coefficient of variation for torque is between 0.11 and 0.19. It is widely accepted that when the coefficient of variation exceeds 0.15, the patterns embedded in the data variations are likely to be obscured [39]. This indicates that the fluctuations in torque have significantly compromised its reliability.

**Figure 6:**
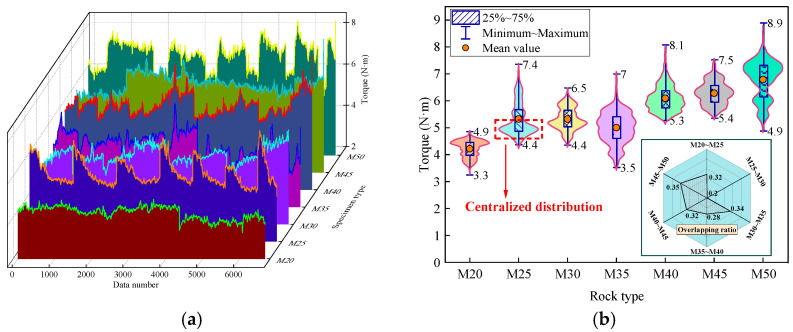
Fluctuation characteristics of torque for different samples: (**a**) Fluctuation characteristics of torque. (**b**) Coefficient of variation for torque.

Figure 6 illustrates the torque fluctuation characteristics for different types of samples. From Figure 6a, it is evident that data fluctuations lead to significant overlap in torque for various rocks, with the overlap increasing as the sample strengths become closer. For instance, in the cases of the M40 and M45 samples, the torque ranges are 5.3 N·m to 8.0 N·m and 5.3 N·m to 7.6 N·m, respectively. Notably, the torque range of M45 falls completely within that of M40, which is clearly counterintuitive. This study utilizes the torque overlap calculation equation presented in Equation (4):

(4) $$k=\frac{\mathrm{Countif}\left\{R\geq \min(S_{fc\geq 80\%})\right\}}{\mathrm{Count}\left\{R\cup S\right\}}$$

Herein, the functions Count and Countif are used for counting and conditional counting, respectively. *R* and *S* represent the torque data sequences of the low-strength and high-strength samples, respectively. *S_fc_* is a data sequence where the relative frequency of torque for high-strength samples exceeds 80%, while min represents the minimum value function.

From Figure 6b, it can be seen that when the strength difference is less than 5 MPa, the torque overlap rate ranges from 20% to 35%, with an average overlap rate of 30%. However, despite the high torque overlap rate, torque exhibits a distinct concentrated distribution characteristic at different rock strengths. This suggests that the torque within the concentrated distribution range is more sensitive to changes in rock strength. Utilizing the torque within this range to predict rock strength could significantly enhance the accuracy of the predictions.

### 3.3. Response Characteristics of Average Torque to Strength Variations

**Figure 7:**
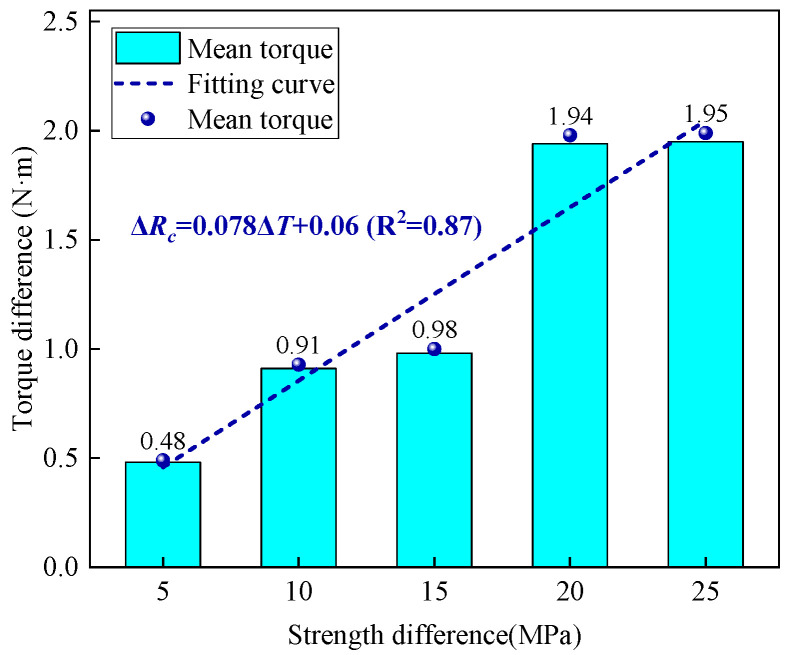
The variation of torque difference with strength difference.

Figure 7 illustrates the relationship between strength difference and torque difference. It can be observed that as the strength difference increases, the torque difference also generally increases. Furthermore, when the strength difference exceeds 15 MPa, the torque difference is twice that of the torque difference when the strength difference is less than 15 MPa. The slope of the fitted curve between strength difference and torque difference is 0.078 N·m, with a determination coefficient of 0.87, indicating a significant linear relationship between the two.

Additionally, when the strength difference increases from 10 MPa to 15 MPa, the increment in torque difference is 0.07 N·m. In contrast, when the strength change varies from 20 MPa to 25 MPa, the increment in torque difference is only 0.01 N·m. In other words, relying solely on the increment of torque difference is insufficient for accurately determining rock strength. For instance, suppose that the torque difference between two types of rocks, A and B, is both 1.94 N·m. If the strength of rock A is 5 MPa, then the torque difference cannot determine whether the strength of rock B is 25 MPa or 30 MPa.

## 4. Unimodal Distribution Characteristics of Torque Frequency for Single-Strength Rocks

### 4.1. Concentrated Distribution Behavior of Torque Frequency for Rocks with Different Strengths

**Figure 8:**
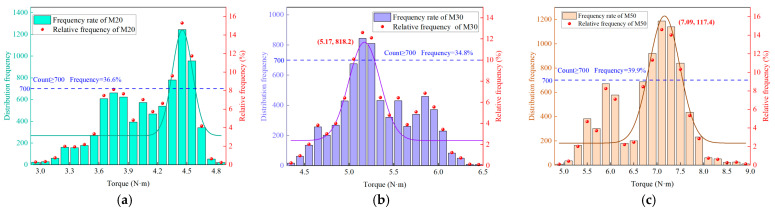
Distribution characteristics of torque frequency: (**a**) M20. (**b**) M30. (**c**) M50.

Data fluctuations lead to the overlap of torque data for rocks with different strengths, reducing the accuracy of strength identification. However, the concentrated distribution characteristic of torque for different strength rocks provides an opportunity to address this issue. Figure 8 illustrates that the torque frequency distributions of rocks with varying strengths all exhibit a unimodal pattern, which is a universal characteristic. In this study, the range corresponding to the frequency peak is defined as the characteristic interval. When the frequency threshold is set at 700, the relative frequency of data within this interval ranges from 34.2% to 48.9% (with an average of 40.3%), which constitutes the majority of the total torque data. Moreover, the overlap rate of the torque data within the characteristic interval is significantly reduced by approximately 20%. For instance, while the complete torque data for the M25 and M30 samples originally overlapped, their respective torque ranges within the characteristic interval are 4.7 N·m to 5.1 N·m and 5.2 N·m to 5.3 N·m, successfully eliminating the overlapping range.

To address the issue of insufficient relative frequency and low data utilization within the characteristic interval of the concentrated distribution, this study proposes a segmented mean method. This method segments the original torque sequence *R*(*x*) into different parts and calculates the mean torque for each segment, reconstructing it into a new sequence *S*(*x*). The aim is to enhance the degree of data concentration and utilization. Assuming that *R*(*x*) contains *L* data points and each sub-interval contains *m* data points, Equation (5) expresses the data in the segmental torque sequence *S*(*x*):

(5) $${\bar{x}}_{ij}=\frac{1}{m}\sum_{j=1}^{m}x_{ij}(i=1,2,\cdots ,L/m)$$

where *i* is the interval count, *j* is the data count within each sub-interval, *x_ij_* is the data in the original torque sequence, and ${\bar{x}}_{ij}$ is the data in the segmental torque sequence.

**Figure 9:**
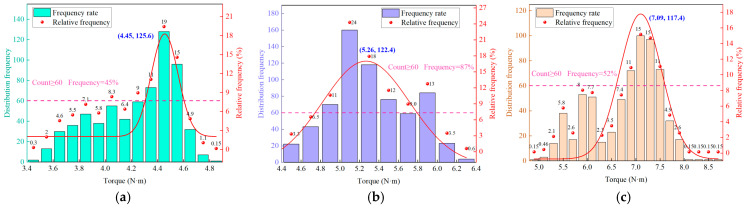
Frequency distribution of segmental torque sequence (*j* = 10): (**a**) M20. (**b**) M30. (c) M50.

In this study, to increase the frequency of torque data within the characteristic intervals, a segmented mean method was proposed for processing the torque data, with *j* set to 10. When the subset length was set to 10, the frequency distribution characteristics of the torque data were most distinct, and the proportion of data within the characteristic intervals was relatively high. The original torque sequences of samples with different strengths are incorporated into Equation (5) to obtain the segmented torque sequences. The frequency distribution of these segmented torque sequences is then calculated, as shown in Figure 9.

As displayed in Figure 9, the frequency distribution remains unimodal, and the boundaries of the characteristic interval remain essentially unchanged when the frequency threshold is set at 60, indicating that this method has a minimal impact on the distribution characteristics. However, the relative frequency of torque within the characteristic interval increases from 40.3% to 66.8%. Additionally, the number of bars for M20, M30, and M45 decreases by 5, 12, and 11, respectively, demonstrating that the segmented mean method significantly enhances data utilization and concentration.

### 4.2. Quantitative Method of the Characteristic Interval of Unimodal Frequency Distribution

The selection of the frequency characteristic interval directly affects the overlap rate of torque data. However, the current methods for determining frequency thresholds are highly subjective, and it is challenging to establish a uniform threshold when there is a significant variation in the torque data of different rocks. Therefore, this study, based on the analysis in Table 4, aims to seek an objective method for determining the characteristic interval.

According to Table 4, the peak torque (*T_p_*) before and after segmentation remains largely consistent. However, the standard deviation of the segmented torque significantly decreases, and the peak torque exhibits an approximately symmetrical distribution in relation to the characteristic interval boundaries. Additionally, the correlation between the standard deviation of the segmented torque and the distance from the boundaries is stronger. Based on this, this study proposes using “*T_p_* ± *σ”* as the quantification criterion for the unimodal frequency characteristic interval (CIS). The peak torque of the frequency fitting curve is determined through differentiation, and the interval boundaries are calculated using Equation (6):

(6) $$T_{p}{|}_{(dF/dT)=0}-\sigma \leq \mathrm{CIS}\leq T_{p}{|}_{(dF/dT)=0}+\sigma$$

**Figure 10:**
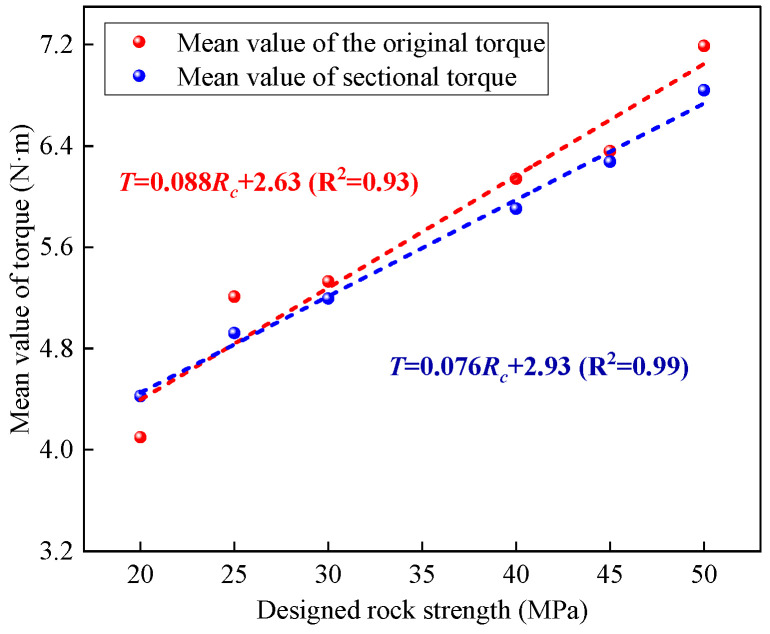
Regression model between average torque and rock strength.

Based on Equation (6), the torque data within the characteristic interval is selected, and the mean is calculated to establish a regression model relating it to rock strength, as shown in Figure 10. The results indicate that there is a significant linear relationship between rock strength and both the original and segmented torque means. Furthermore, the coefficient of determination for the segmented torque mean increases to 0.99 (compared with 0.93 for the original mean), suggesting a stronger correlation with rock strength.

### 4.3. Effectiveness of Rock Strength Identification Based on Torque Within the Characteristic Interval

To validate the effectiveness of torque within the characteristic interval for rock strength identification, this study employs a support vector machine (SVM) suitable for small sample problems for prediction. This method achieves effective sample classification and maximization of the margin by finding the optimal classification hyperplane [40]. The original torque and the segmented characteristic interval torque are used as inputs, while rock strength serves as the output. The dataset is divided into training and testing sets in a ratio of 70% to 30%. To mitigate the risk of overfitting, all available MWD experimental data were incorporated into the model training process. This approach helps minimize the potential for overfitting to noise by fully utilizing the entire variability present in the data. Additionally, a five-fold cross-validation scheme was adopted to evaluate model performance; this method ensures that the model is validated across different data partitions.

**Figure 11:**
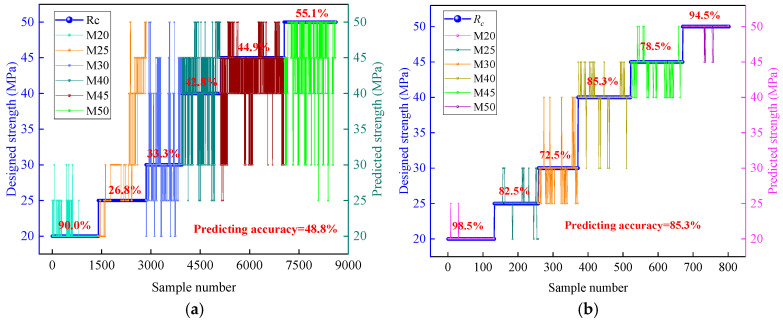
Prediction accuracy of rock strength: (**a**) Prediction accuracy using original torque. (**b**) Prediction accuracy of segmental torque within CIS.

A drilling identification model is established based on the Fitcsvm algorithm, resulting in the strength prediction outcomes shown in Figure 11. From the figure, when predicting rock strength using the original torque, the overall accuracy is only 48.8%, with the M25 sample being the lowest at 28.6% and the M20 sample the highest at 90%. The high misclassification rate is primarily due to severe overlap in the torque data. In contrast, when predicting using the torque within the characteristic interval, the overall accuracy improves to 85.3%, with all samples except for M30 and M45 achieving accuracies exceeding 82%. Compared with the original data, the segmented characteristic interval method enhances prediction accuracy by 36.5%.

Based on the model’s prediction results, this paper presents the confusion matrices shown in Table 5 and Table 6. Using these confusion matrices, we calculate the accuracy, recall, and specificity of the original torque data and the characteristic interval torque data for rock strength prediction. For the original torque data, the model’s predicted accuracy is 49.3%, with recall rates for each class being 90%, 27%, 33%, 43%, 45%, and 55% and specificity rates being 97%, 93%, 89%, 80%, 87%, and 94%. For the characteristic interval torque data, the model’s predicted accuracy is 85.4%, with recall rates for each class being 98%, 83%, 73%, 85%, 79%, and 95% and specificity rates being 99%, 96%, 97%, 95%, 96%, and 99%.

Comparing the performance of the SVM prediction model on the original torque data versus the characteristic interval torque data, we find that the accuracy of the model with the original torque data is only 49.3%, indicating a weak overall discrimination capability. The significant variation in recall rates across different categories (27% to 90%) suggests that the model exhibits a serious phenomenon (failure to identify) for different rock strength categories. Although the specificity is generally high (ranging from 80% to 97%), indicating a reasonable ability to exclude negative samples, the low recall rates limit the practical application value of the model. In contrast, the characteristic interval torque data significantly enhances the model’s accuracy to 85.4%, with recall rates (ranging from 73% to 98%) and specificity rates (ranging from 95% to 99%) both remaining at elevated levels. This indicates that the model performs more robustly and equitably in identifying positive samples and excluding negative samples. It suggests that the frequency distribution method effectively strengthens the discriminatory relationship between torque data and rock strength categories, substantially improving the overall classification performance and reliability of the model.

**Figure 12:**
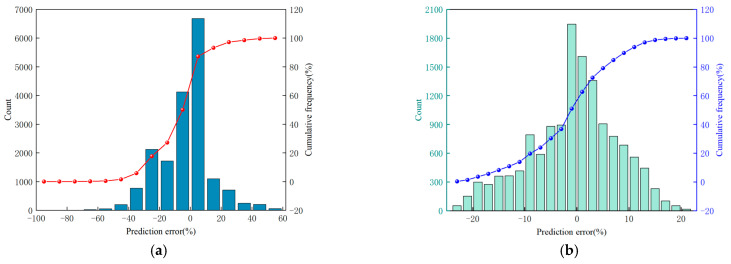
Prediction accuracy of PSO−BP model on rock strength: (**a**) Prediction accuracy using original torque. (**b**) Prediction accuracy of segmental torque within CIS.

Furthermore, this study constructed the PSO−BP model to predict rock strength, aiming to further validate the improvement in prediction accuracy achieved by the concentrated frequency distribution method. The numbers of neurons in the input, hidden, and output layers of the PSO-BP model were set to 1, 4, and 1, respectively. During the training process, the root mean square error (RMSE) was used as the performance evaluation metric. Using the original torque data and the torque data processed by the concentrated frequency distribution method as input datasets and the designed rock strength as the output dataset, the training set and testing set were divided in a 70% to 30% ratio. The prediction results of the PSO−BP model for rock strength are shown in Figure 12.

As shown in Figure 12, when using the original torque as input, the slope of the fitting curve between the designed strength and the predicted strength was 0.69. The prediction errors were mainly distributed in the range of 0–40%, with a maximum error of up to 70% and an average error of approximately 11.8%. In contrast, after screening the torque data using the concentrated frequency distribution method, the slope of the fitting curve increased to 0.93, the concentration range of prediction errors narrowed to 0–20%, and the average prediction error decreased from 11.8% to 6.4%, representing a reduction of 4.5%. These results demonstrate that the concentrated frequency distribution method significantly enhances the accuracy and stability of rock strength identification while drilling.

## 5. Multimodal Distribution Characteristics of Torque Frequency for Composite Rocks

The roadways in coal mines commonly feature composite strata, and the MWD technology involves drilling through various lithological strata. Each stratum is associated with a specific characteristic interval of frequency distribution for torque. Consequently, the frequency distribution of the overall borehole’s torque data will exhibit multimodal characteristics. By analyzing factors such as the number of peaks, characteristic intervals, and torque frequency, we can make predictions regarding the lithology, strength, and thickness of the strata.

### 5.1. Concentrated Distribution Behavior of Torque Frequency for Composite Rocks

**Figure 13:**
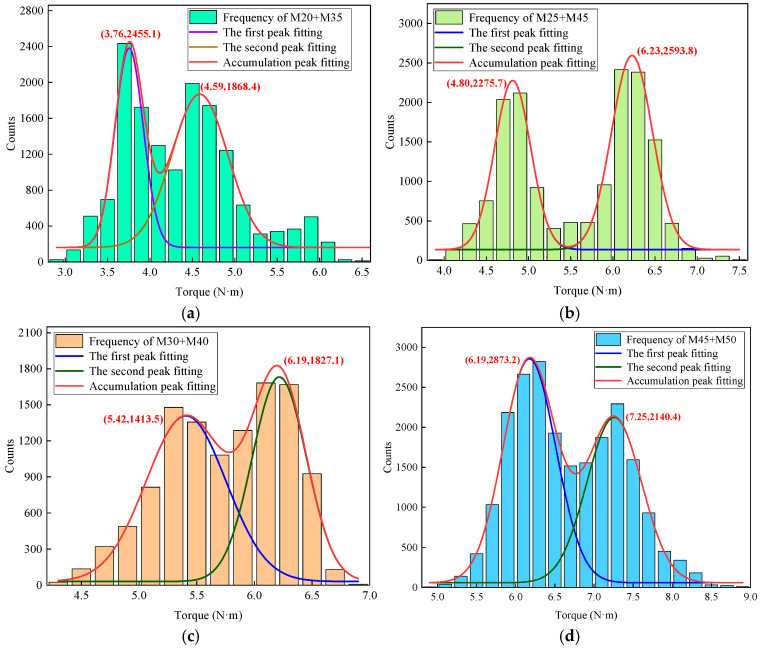
Distribution characteristics of torque frequency for samples 1~4: (**a**) Sample 1. (**b**) Sample 2. (**c**) Sample 3. (**d**) Sample 4.

The roof strata of coal mine roadways are often composed of mixed rock layers, and the drilling parameters exhibit corresponding frequency distributions due to the different rock layers. The overall drilling parameters for each borehole display a multimodal characteristic. By analyzing the number of peaks, characteristic intervals, and frequency counts, one can infer the lithology, strength, and thickness of the roof strata. Figure 13 presents the torque frequency distributions for four groups of samples, revealing that the torque data for different samples exhibit a bimodal distribution. Each peak corresponds to a specific type of rock, with lower peak torques indicating lower rock strength. A Gaussian function is employed for multimodal fitting to obtain the cumulative curve, and the peak torques are statistically compiled in Table 7.

**Figure 14:**
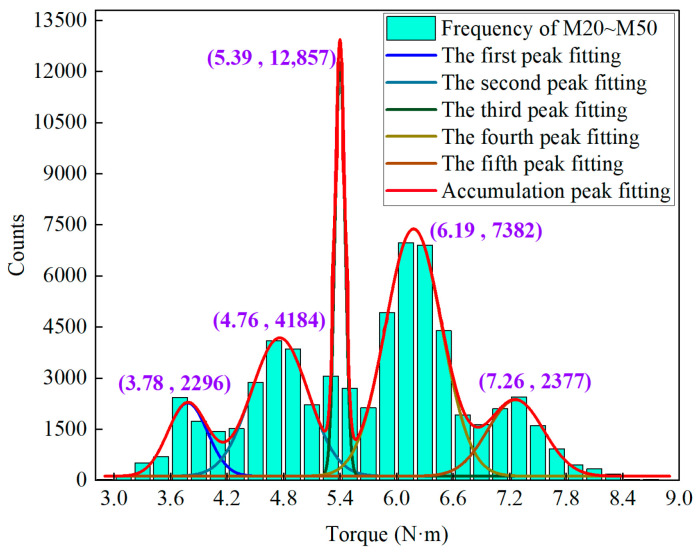
Multimodal characteristics of combined torque data for four groups of rock samples.

To validate the feasibility of identifying composite rock layers using the multimodal frequency feature, the torque data from four groups of samples were mixed, resulting in the distribution shown in Figure 14. As observed, the torque frequency exhibits five distinct peaks, corresponding to five types of rock. However, the actual number of rock types is seven, with the missing rock types being M35 and M40. This phenomenon is attributed to the overlapping frequency peak values of M25/M35 and M40/M45. Additionally, the peak torque values are 3.78, 4.76, 5.39, 6.19, and 7.26 N·m. As the strength difference of the composite samples increases, the average distance between the peaks rises from 0.9 N·m to 2.44 N·m, indicating that greater differences in rock layer strength lead to more pronounced multimodal characteristics, thus facilitating the identification of lithology and strength.

**Figure 15:**
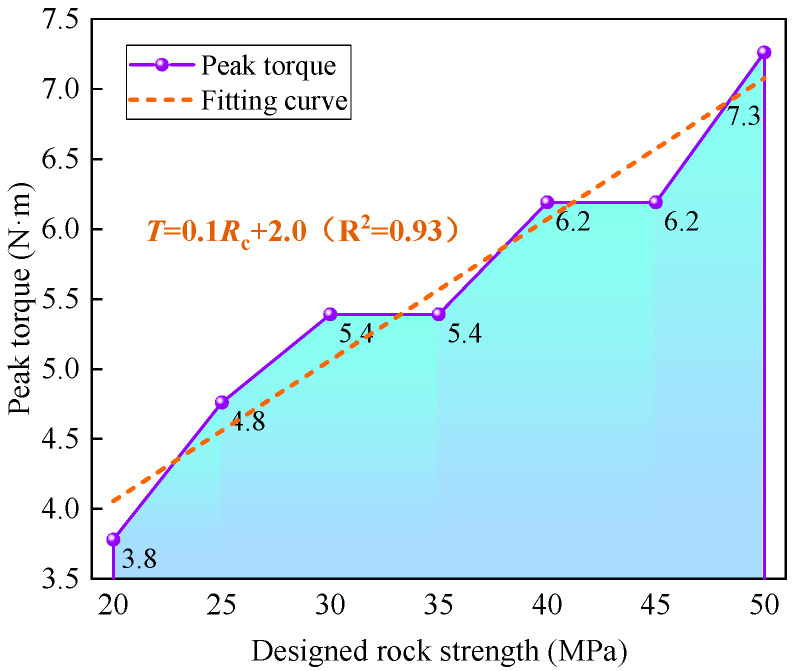
Relationship between peak torque and rock strength.

The relationship between peak torque and rock strength is illustrated in Figure 15. As depicted, the fitting equation for rock strength and peak torque can be expressed as *T_p_* = 0.1*R_c_* + 2.0, exhibiting a determination coefficient of 0.93, indicating an incremental relationship of 0.1 N·m/MPa. Consequently, when the strength difference exceeds 5 MPa, peak torque demonstrates a commendable lithology identification effect and can serve as an effective indicator for predicting rock strength.

### 5.2. Quantitative Method of the Characteristic Interval of Multimodal Frequency Distribution

**Figure 16:**
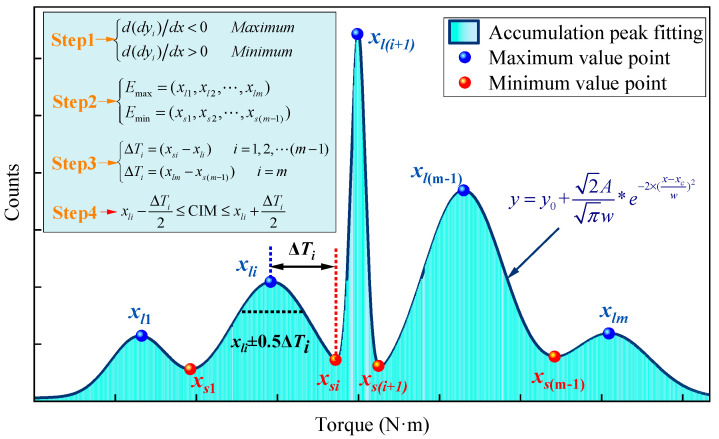
Principle of determining characteristic interval of composite rocks using extremum method.

Unlike the single-peak distribution of frequency quantification, multimodal frequency distributions exhibit multiple extreme points, making it impossible to directly determine the peak torque using the first derivative. Therefore, this study proposes using the extremum method to solve for the frequency multimodal characteristic intervals, the principle of which is illustrated in Figure 16.

The calculation steps for the frequency multimodal characteristic intervals based on the extremum method are as follows: First, the cumulative peak fitting equation of the torque frequency distribution of composite rock layers is obtained using Gaussian fitting [41]. Next, the first derivative of this equation is solved to determine the locations of the extreme points, and the type of each extreme point (maximum or minimum) is identified based on the sign of the second derivative. Then, the extreme points *x_i_* are categorized into a sequence of maxima *E_l_* and a sequence of minima *E_s_*. After arranging them in ascending order, the distances *∆T_i_* between each maximum and its adjacent minimum are calculated in order. Finally, based on the peak torque and the distances of the extreme points, the characteristic intervals CIM corresponding to each frequency peak are determined, as demonstrated in Equation (7):

(7) $$x_{li}-\frac{\Delta T_{i}}{2}\leq \mathrm{CIM}\leq x_{li}+\frac{\Delta T_{i}}{2}$$

### 5.3. Thickness of Strata Calculation Model Based on Torque Frequency

Previous studies have focused on the relationship between peak torque and average values within characteristic intervals and rock strength but have not fully explored the rock layer information contained within torque frequency. Theoretically, at a fixed sampling frequency, the torque frequency is closely related to rock strength and thickness: the rock layer thickness and penetration rate together determine the frequency value, while the on-site penetration rate is primarily controlled by the rock strength. Therefore, this study aims to statistically analyze the torque frequency within the characteristic intervals, eliminate frequency differences caused by strength variations, and invert the thickness of each rock layer in conjunction with borehole depth. The specific steps are as follows.

Assuming the sampling frequency of the data monitoring system is *f*, the difference in torque frequency *Q_R_* caused by varying rock strength within the *i*-th characteristic interval can be expressed as shown in Equation (8):

(8) $$Q_{Ri}=f/v$$

Numerous studies have indicated that there is an inherent functional relationship between rock strength and penetration rate [42]. Let us denote this relationship as *k*. By substituting this into Equation (8), we can derive the relationship between rock strength and the difference in torque frequency, as given in Equation (9):

(9) $$Q_{Ri}=kf/R_{c}$$

The torque frequency *Q_Hi_* within the *i*-th characteristic interval, which is primarily influenced by the thickness of strata, can be mathematically expressed as Equation (10).

When the torque frequency within the *i*-th characteristic interval is *Q_i_*, the torque frequency *Q_Hi_* mainly related to the rock layer thickness within that interval can be expressed as in Equation (10):

(10) $$Q_{Hi}=Q_{i}-Q_{Ri}$$

Finally, based on *Q_Hi_* and borehole depth *H*, we obtain the thickness estimation formula for different rock layers, as shown in Equation (11):

(11) $$h_{i}=(Q_{Hi}/\sum_{i=1}^{m}Q_{Hi})\times H$$

### 5.4. Identification Strategy of Strata Information Based on Frequency Distribution Characteristics

**Figure 17:**
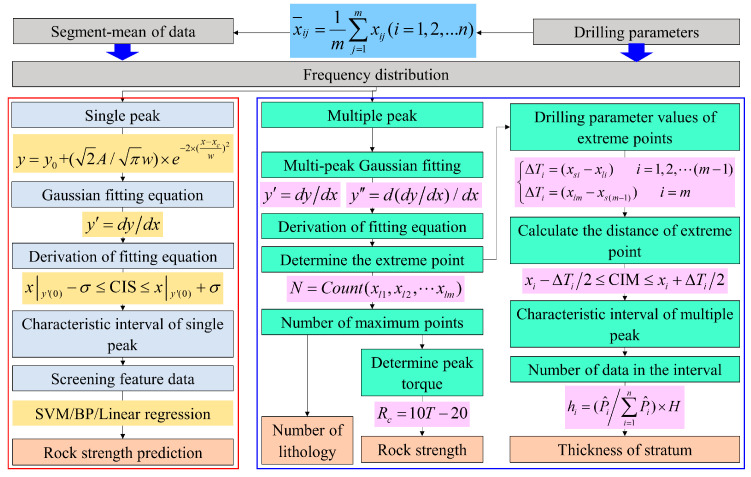
Identification strategy of strata information based on frequency distribution characteristics.

In this study, the distribution characteristics of torque frequency are utilized to identify rock strength during drilling. However, the purpose of this study is to take torque data as an example to reveal the general law of drilling parameters. Therefore, for other drilling parameters such as thrust, rotational speed, and penetration rate, it also has frequency distribution characteristics similar to torque data. To facilitate its application, a process for identifying strata information based on the frequency distribution of drilling parameters is presented in Figure 17.

For single-strength rock layers, the segmental mean method is used to process the drilling parameters. Then, the characteristic intervals are determined through frequency distribution and fitting equations. Based on the drilling parameters within these characteristic intervals, either a support vector machine (SVM) or a backpropagation (BP) neural network is established to predict rock strength. For composite rock layers, the identification of rock types and quantities is achieved using the extremum method based on multimodal frequency fitting. The peak torque is utilized to predict strength, and the frequency of parameters within the characteristic intervals is further employed to estimate rock layer thickness.

This paper proposes a method to identify rock strength based on the frequency distribution characteristics of torque data, but there are the following limitations:

(1) The current study focuses solely on torque data and does not incorporate other drilling parameters (such as thrust, rate of penetration, and vibration). In future work, we plan to introduce integrated multi-sensor data analysis methods to improve the accuracy and robustness of rock strength identification.
(2) The laboratory tests adopted a “constant rotational speed–constant penetration rate” control mode. However, in field drilling, rotational speed and penetration rate are often adjusted dynamically according to formation changes, which in turn affects the response characteristics of parameters such as torque and thrust. Therefore, our next step will focus on investigating the coupling mechanisms between control parameters and drilling parameters.
(3) Due to limitations in field-testing conditions, the robustness of the proposed method when applied beyond the tested strength range has not yet been fully validated. We will actively promote industrial field trials in the next phase, compare differences between laboratory and field drilling data, and refine the existing models and methods to make them more suitable for practical engineering environments.

## 6. Conclusions

This study established a characteristic interval quantification model for single-strength rocks and composite rock layers based on torque frequency distribution characteristics and proposed methods for identifying the number, strength, and thickness of rock layers. The main conclusions are as follows:

(1) The equation between penetration rate and torque can be expressed as *T_c_* = *T_p_* − 0.86*v* + 6.3, and the average torque overlap rate for rocks with adjacent strengths (Δ*R_c_* < 5 MPa) is 34%.
(2) The torque for single-strength rock layers exhibits a unimodal distribution. The characteristic interval model CIS = *T_p_*|_(*dF*/*dT*) = 0_ ± *σ* can improve the strength prediction accuracy from 48.8% to 85.3%.
(3) The torque for composite rock layers shows a multimodal distribution, and strength can be estimated using *R_c_* = 10*T_p_*−20. The multimodal interval model CIM = *x_li_* ± 0.5∆*x_i_* and the equation of strata thickness *h_i_* = (*Q_Hi_*/$\sum_{i=1}^{m}Q_{Hi}$) × *H* enable effective prediction of rock layer thickness.

## Figures and Tables

**Table 1:** Material ratio of rock samples.

Sample Type	Sand (kg/m^3^)	Water (kg/m^3^)	Cement (kg/m^3^)	Water Reducer (kg/m^3^)
M20	1450	310	400	0
M25	1450	310	425	0
M30	1450	310	460	0
M35	1450	310	500	0
M40	1450	310	550	0
M45	1450	310	620	0
M50	1350	200	600	6.24

**Table 2:** Strength of different composite rock samples.

Sample Number	Designed Strength (MPa)	Measured Strength (MPa)	Strength Difference (MPa)
#1	20 + 35	17.85 + 33.46	∆ = 15.61
#2	45 + 25	43.38 + 24.69	∆ = 18.69
#3	30 + 40	28.41 + 41.52	∆ = 13.11
#4	45 + 50	44.38 + 51.26	∆ = 6.88

**Table 3:** Drilling test plan.

Specimen Number	Borehole Number	Rotational Speed	Penetration Rate
1	S1-B1~S1-B9	300~600 rpm (∆*n* = 50 rpm)	2~6.5 mm/s (∆*v* = 0.5 mm/s)
2	S2-B1~S2-B9		
3	S3-B1~S3-B9		
4	S4-B1~S4-B9		

**Table 4:** Comparison of characteristic intervals of pre- and post-segmentation.

Segmentation	Data Characteristics (N·m)	M20	M25	M30	M40	M45	M50
*j* = 1	Characteristic interval	4.3~4.6	4.7~5.2	5.1~5.3	5.4~6.4	6.2~6.5	6.8~7.6
	Peak torque	4.45	4.9	5.15	6.0	6.35	7.2
	Boundary distance	[0.2,0.2]	[0.2,0.3]	[0.1,0.2]	[0.4,0.4]	[0.2,0.2]	[0.4,0.4]
	Standard deviation	0.39	0.43	0.47	0.51	0.54	0.56
*j* = 10	Characteristic interval	4.2~4.6	4.6~5.2	4.8~5.9	5.5~6.3	5.7~6.9	6.8~7.6
	Peak torque	4.45	4.9	5.3	5.9	6.3	7.2
	Boundary distance	[0.3,0.3]	[0.3,0.3]	[0.5,0.6]	[0.4,0.4]	[0.6,0.6]	[0.4,0.4]
	Standard deviation	0.31	0.41	0.45	0.47	0.53	0.51

Notes: (1) Peak torque refers to the torque at the peak of the frequency fitting curve. (2) Boundary distance is the distance between the peak torque and the boundaries of the characteristic interval.

**Table 5:** The confusion matrix of the prediction model under the original torque data.

Confusion Matrix		Predicted Strength					
		20	25	30	40	45	50
Designed strength	20	1262	167	53	0	0	0
	25	135	388	382	27	3	8
	30	5	475	363	178	119	80
	40	0	211	214	502	746	294
	45	0	174	64	305	869	326
	50	0	34	16	159	198	869

**Table 6:** The confusion matrix of the prediction model under the characteristic interval torque data.

Confusion Matrix		Predicted Strength					
		20	25	30	40	45	50
Designed strength	20	130	8	0	0	0	0
	25	2	104	23	0	0	0
	30	0	14	82	6	0	0
	40	0	0	8	127	27	0
	45	0	0	0	17	117	7
	50	0	0	0	0	5	125

**Table 7:** Peak torque for rock samples #1~#4.

Sample Group	Sample #1	Sample #2	Sample #3	Sample #4
First peak torque/N·m	3.76 (M20)	4.80 (M25)	5.42 (M30)	6.19 (M45)
Second peak torque/N·m	4.59 (M35)	6.23 (M45)	6.19 (M40)	7.25 (M50)

## Data Availability

The data presented in this study are available on request from the corresponding author.

## Abbreviations

The following abbreviations are used in this manuscript:

CIM	Characteristic intervals of multimodal frequency distribution
CIS	The characteristic interval of unimodal frequency distribution
*c_n_(t)*	The ensemble mean value of each IMF
*Count*	Counting function
*Countif*	Conditional counting function
*c_v_*	Coefficient of variation
EEMD	Ensemble empirical mode decomposition
EMD	Empirical mode decomposition
*E_l_*	The sequence of maximum value point
*E_s_*	The sequence of minimum value point
*f*	The sampling frequency of the data monitoring system (Hz)
Fitsvm	Cross-validation support vector machine
*g_m_(t)*	Gaussian white noise signals
*H*	Drilling depth (m)
*h_i_*	An approximate thickness of different strata (m)
*i*	Sub-interval count
IMFs	Multiple intrinsic mode functions
*j*	Data count within each sub-interval
*k*	Inherent functional correlation between rock strength and penetration rate
*L*	Original torque sequence contains the number of data points
*m*	Each sub-interval contains the number of data points
min	The minimum value function
*n*	The number of maxima
*Q_Hi_*	Torque frequency influenced by the thickness of strata within the *i*-th characteristic interval
*Q_Ri_*	The torque frequency difference caused by strength variations within the *i*-th characteristic interval
*R_l_*	Torque data sequences of the low-strength samples
*R(x)*	Original torque sequence
*S*(*x*)	Segmental torque sequence
*S_h_*	Torque data sequences of the high-strength samples
*S_fc_*	Torque data sequence where the frequency exceeds 80% for high-strength samples
SVM	Support vector machine
*T*	Torque (N·m)
*T_c_*	The torque obtained after eliminating the influence of penetration rate (N·m)
*T_r_*	The original torque (N·m)
*T_p_*	Peak torque (N·m)
*v*	Penetration rate (mm/s)
*x*(*t*)	Original signal
*x_i_*	The positions of extreme points
*x_ij_*	The data in the original torque sequence
${\bar{x}}_{ij}$	The data in the segmental torque sequence
*x_li_*	The positions of maximum value point
*x_si_*	The positions of minimum value point
*μ*	The mean value of the data
*σ*	The standard deviation of the data
*ρ*	Overlap rate of torque data for adjacent strength samples
*∆R_c_*	Strength changes (MPa)
*∆T*	Torque variation (N·m)
*∆x_i_*	The distance between each peak and trough in the sequence
